# A systematic pan-cancer analysis identifies TRIM28 as an immunological and prognostic predictor and involved in immunotherapy resistance

**DOI:** 10.7150/jca.86742

**Published:** 2023-09-04

**Authors:** Zhi Shang, Xinqiang Wu, Shengfeng Zheng, Yaru Wei, Zhe Hong, Dingwei Ye

**Affiliations:** 1 Department of Urology, Fudan University Shanghai Cancer Center, Shanghai, China.; 2 Department of Oncology, Shanghai Medical College, Fudan University, Shanghai, China.; 3 Shanghai Genitourinary Cancer Institute, Shanghai, China.; 4 Institute for translational brain research, Fudan University, Shanghai, China.

**Keywords:** TRIM28, pan-cancer analysis, immunotherapy, prognostic biomarker

## Abstract

Tripartite motif-containing protein 28 (TRIM28), as a transcriptional cofactor, has pleiotropic biological effects, such as silencing genes, promoting cellular proliferation and differentiation, and facilitating DNA repair. It is reported that TRIM28 is also correlated with immune infiltration in liver cancer that highlights an unnoticed function of TRIM28 in immune system. However, the prognostic and immunotherapeutic role of TRIM28 in human cancer has not been elucidated. In this study, we conducted a systematic pan-cancer analysis and partial experimental validation of TRIM28 as an immunological and prognostic predictor and its involvement in immunotherapy resistance. We found that TRIM28 expression was higher in various tumor tissues than in normal tissues. Higher TRIM28 expression was associated with poorer prognosis in multiple cancers. The expression of TRIM28 was positively correlated with the presence of T cells, macrophages and neutrophils, and TRIM28 also promoted the infiltration of a series of immune cell. Moreover, TRIM28 affected a wide range of cancer-related scores, and the abnormal expression of TRIM28 was also involved in tumor mutational burden, drug sensitivity, and microsatellite instability in cancer. The results suggest that TRIM28 is a potentially valuable immune response indicator and a molecular biomarker for predicting the prognosis of cancer patients.

## Introduction

Cancer is the most common cause of death worldwide and greatly compromises quality of life in all countries, but unfortunately, there is currently no absolute cure. The number of new cancer cases and deaths is expected to rise to 19.3 million and 10.0 million, respectively, by 2020 1. Because of this situation, cancer is an increasingly heavy burden on society as a whole 1. Immunotherapy for cancer, especially immune checkpoint blockade therapy, has gained popularity for its ability to increase the survival time of cancer patients 2. However, the effect of immunotherapy is still far from satisfactory. Therefore, it is urgent to study the pathogenesis of cancer and identify effective therapeutic targets.

Because of the ongoing development and improvement of public databases, it is possible to conduct pan-cancer analyses of target genes and assess their relevance to clinical prognosis and associated signaling pathways, which contributes to the development of novel therapeutic methods for cancer treatment 3.

Tripartite motif-containing protein 28 (TRIM28) was first reported in 1996 4, and it is considered to be a significant regulator of human carcinogenesis 5-7. The amino terminus of TRIM28 contains a ring-finger domain, a coiled-coil region, four conserved domains, and a B-box type 1 and a B-box type 2, which are collectively referred to as the RBCC domain 8, 9. As a transcriptional cofactor, TRIM28 has pleiotropic biological effects, such as silencing genes, promoting cellular proliferation and differentiation, and facilitating DNA repair 10. A previous study revealed that TRIM28 promotes T-cell activation 11 and tolerance 12. TRIM28 upregulation is reported to be correlated with poor prognosis in gastric cancer 13. TRIM28 has also been recently found to protect its family member TRIM24 from SPOP-mediated degradation, thereby aggravating the progression of prostate cancer (PCa) 14. Furthermore, TRIM28 can serve as an E3 ligase to participate in protein ubiquitination and degradation, and the p53 protein is also a substrate of TRIM28 10, 15. Although TRIM28 is increasingly being found to have important effects on cancer, its effect on the immune system and its relationship to immunotherapy resistance remain poorly understood, and the function and clinical significance of TRIM28 in the human pan-cancer context have not been systematically investigated.

In the present study, we thoroughly analyzed the features of TRIM28 based on a variety of cancer databases to explore the link between TRIM28 and the immune system as well as its possible contribution to immunotherapy resistance. Our study highlights the multifaceted effects of TRIM28 on multiple cancers, providing a possible theoretical basis and a new target for the treatment of cancer.

## Materials and Methods

### TRIM28 expression in human cancers

RNA sequencing data for thirty-three human cancers in the Genotype Tissue Expression (GTEx) and TCGA databases were obtained from the UCSC XENA (https://xenabrowser.net/datapages/) website in TPM format and were processed uniformly via the Toil process. As the first step, we compared the expression of TRIM28 in the Tumor Immune Estimation (TIMER, http://timer.cistrome.org/) database, which is based on TCGA data for various types of cancer. The Wilcoxon rank-sum test in R software was utilized to analyze gene expression differences. Then, ggplot2 (version 3.3.3) was used to visualize gene expression differences. GEPIA2 (http://gepia2.cancer-pku.cn/) is a web-based tool that uses the TCGA and GTEx databases to deliver fast and flexible functionalities. The "Stage plot" module was selected, "TRIM28" was used as the input term, and the sort of malignancies we picked in "Datasets Selection" was chosen to determine the relationship between the expression level of TRIM28 and the pathological tumor stages. TNM plotter (https://tnmplot.com/analysis/) was used to investigate differences in TRIM28 expression between tumor, metastatic, and normal tissues 16.

### Analysis of survival prognosis

With the customizable function, GEPIA2 (http://gepia2.cancer-pku.cn/) is used to explore data in the TCGA and GTEx databases. The "Survival analysis" module was used to examine the relationship between TRIM28 expression and clinical outcomes in human cancers, and the median expression level of TRIM28 was selected as the group cutoff. Overall survival (OS) data, Disease-free survival (DFS) data, and survival curves were acquired via GEPIA2. The survival analysis employed the log-rank test. The effect of TRIM28 expression on patient survival was estimated using the hazard ratio (HR).

### Analysis of genetic alteration

Due to the integration of genetic alteration data, an online tool (https://www.cbioportal.org/) was used to study TRIM28 alterations in human cancers 17. The “Cancer Type Summary” module was chosen to investigate the TRIM28 alteration landscape across cancers. The "Mutation" module was used to generate a mutation site plot for TRIM28. To distinguish the relationship between TRIM28 alterations and clinical outcomes, we chose PRAD and UCS as the cancers and divided the patients into the unaltered and altered groups. The survival curves were generated with the “Comparison/Survival” module.

### TRIM28 and the tumor immune microenvironment (TIME)

RNA-Seq expression profiling data in the TIMER database (https://cistrome.shinyapps.io/timer/) were used to analyze immune cell infiltration in tumor tissues. The correlation between immune cell infiltration and TRIM28 expression was analyzed in multiple cancers. The stromal, immune, and ESTIMATE scores for predicting patient prognosis were calculated by the ESTIMATE algorithm in R software. The relationships between these scores and TRIM28 expression were analyzed via the psych package (version 2.1.6) in R software. The correlation coefficient between two factors was calculated using Pearson correlation analysis.

### TRIM28 and the immunotherapy response

The immunotherapy response was predicted using the Tumor Immune Dysfunction and Exclusion (TIDE) database (http://tide.dfci.harvard.edu) 18. The function of TRIM28 in the tumor immunotherapy response was investigated using the Riaz2017_PD1 SKCM cohort. The relationships between overall patient survival and different TRIM28 expression levels and cytotoxic T lymphocyte (CTL) levels were assessed by the T-cell dysfunction score, and gene expression values in T-cell exclusion signatures were described by the exclusion score. Based on the biomarker evaluation of TRIM28's predictive power for OS and response outcomes, the predictive power of TRIM28 was compared with other published biomarkers. Each gene's T-cell dysfunction score was calculated using the Wald test Z score. Standardization of Z scores can improve data comparability by converting data from different metrics into one uniform metric. By using the Z score, different biomarkers were compared for their effects on immunotherapy efficacy.

### TRIM28 expression at the single-cell level

Visualization of the tumor microenvironment using interactive single-cell transcriptomes is possible through a comprehensive web resource called Tumor Immune Single-Cell Hub (TISCH) (http://tisch. comp-genomics.org/) 19. TRIM28 expression at the single-cell level in the BRCA_GSE114727, KIRC_GSE139555, and PAAD_CRA001160 datasets was visualized with the “dataset” module.

### Immunohistochemical staining

The tissue microarray was obtained from Shanghai Zhuolibiotech Company Co., Ltd. The specimens were embedded in paraffin. Serial 4 μm slices were cut, deparaffinized, blocked, and incubated with the primary antibody for an overnight period at 4 °C before being exposed to the secondary antibody that was labeled with horseradish peroxidase. TRIM28 expression was evaluated using the automated VIS DIA VisioMorph System (Visiopharm). Patient tissue specimens and the Ethics approval in this work was reviewed and approved by the ethics committee of Fudan University Shanghai Cancer Centre.

### Statistical analysis

R software (version 3.3.3, http://www.r-project.org) was used for statistical analysis. The Wilcoxon rank-sum test was performed to compare differences between two groups. The Kruskal-Wallis test was conducted to analyze differences among three or more groups. For all tests, *p* < 0.05 was considered statistically significant.

## Results

### TRIM28 expression across cancers

**Figure 1:**
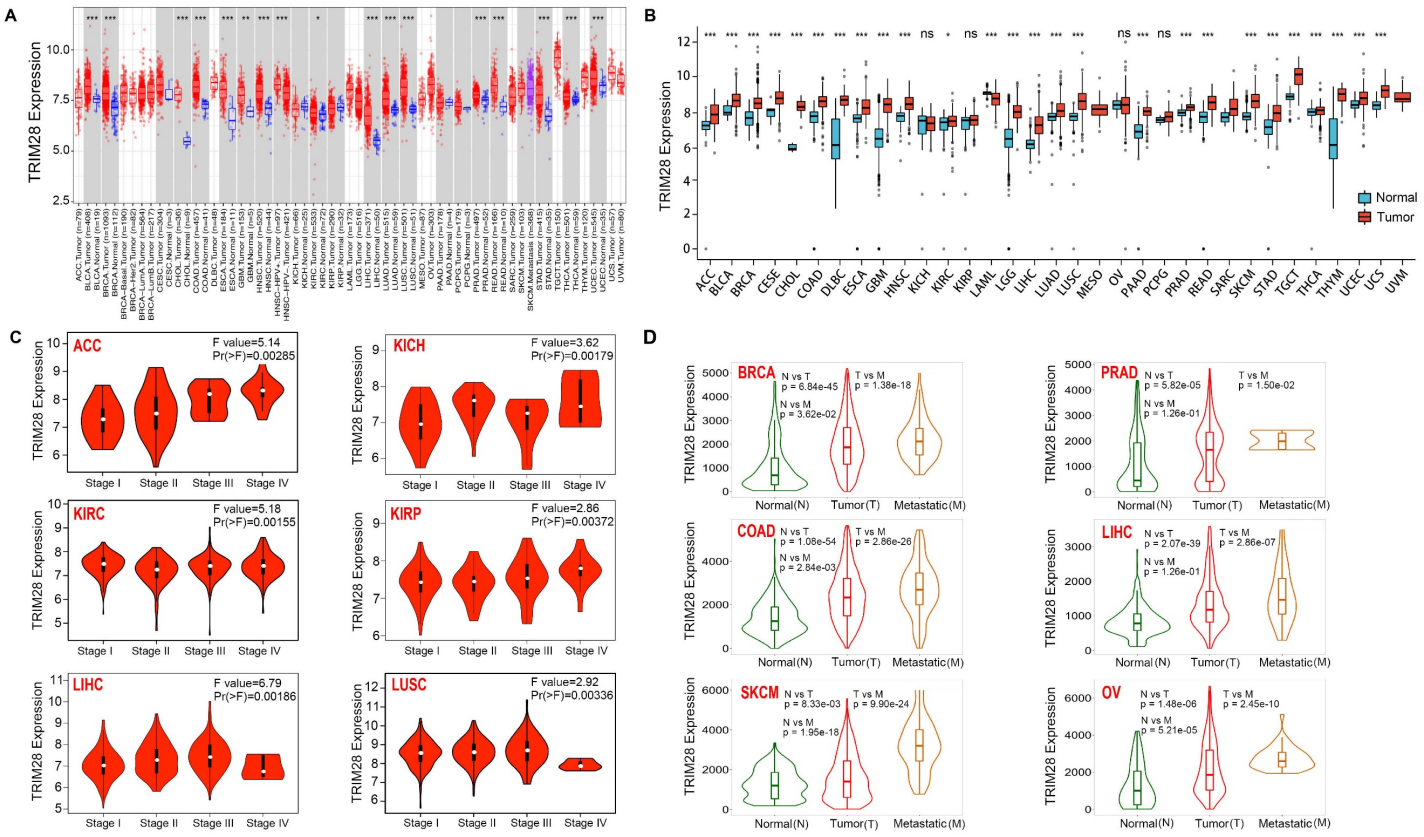
** TRIM28 expression in different pathological stages in various cancers. (A)** TRIM28 expression in tumor and normal tissues via TIMER 2.0. **(B)** TRIM28 expression status in different types of cancers from TCGA and GTEx data. **(C)** Based on the TCGA data, TRIM28 expression levels in different types of cancers (ACC, KICH, KIRC, KIRP, LIHC, LUSC) were analyzed according to pathological stage (stage I, stage II, stage III, and stage IV). **(D)** Differences in TRIM28 expression levels between normal, tumor, and metastatic tissues in BRCA, PRAD, COAD, LIHC, SKCM, and OV. *p < 0.05; **p <0.01; ***p <0.001.

The expression of TRIM28 in tumor tissues of 33 cancer types and the corresponding normal tissues was compared using the TIMER online network tool with data from the TCGA database. As shown in Figure 1A, TRIM28 was widely overexpressed in BLCA, BRCA, ESCA, CHOL, COAD, GBM, LIHC, HNSC, KIRC, LUAD, READ, THCA, LUSC, PRAD, STAD, UCEC 16 types of tumor tissue. We further analyzed the TCGA and GTEx data because several tumors did not have corresponding normal controls in the TCGA database. TRIM28 expression was also significantly increased in ACC, SKCM, DLBC, LAML, CESC, LGG, PAAD, TGCT, THYM, and UCS tumor tissue in addition to the 16 types of tumor tissue mentioned above (Figure 1B). Furthermore, TRIM28 expression was significantly correlated with pathological stage (Figure 1C), and TRIM28 expression in metastatic BRCA, PRAD, COAD, LIHC, SKCM, and OV tumors was higher than that in the corresponding primary tumors (Figure 1D).

**Figure 2:**
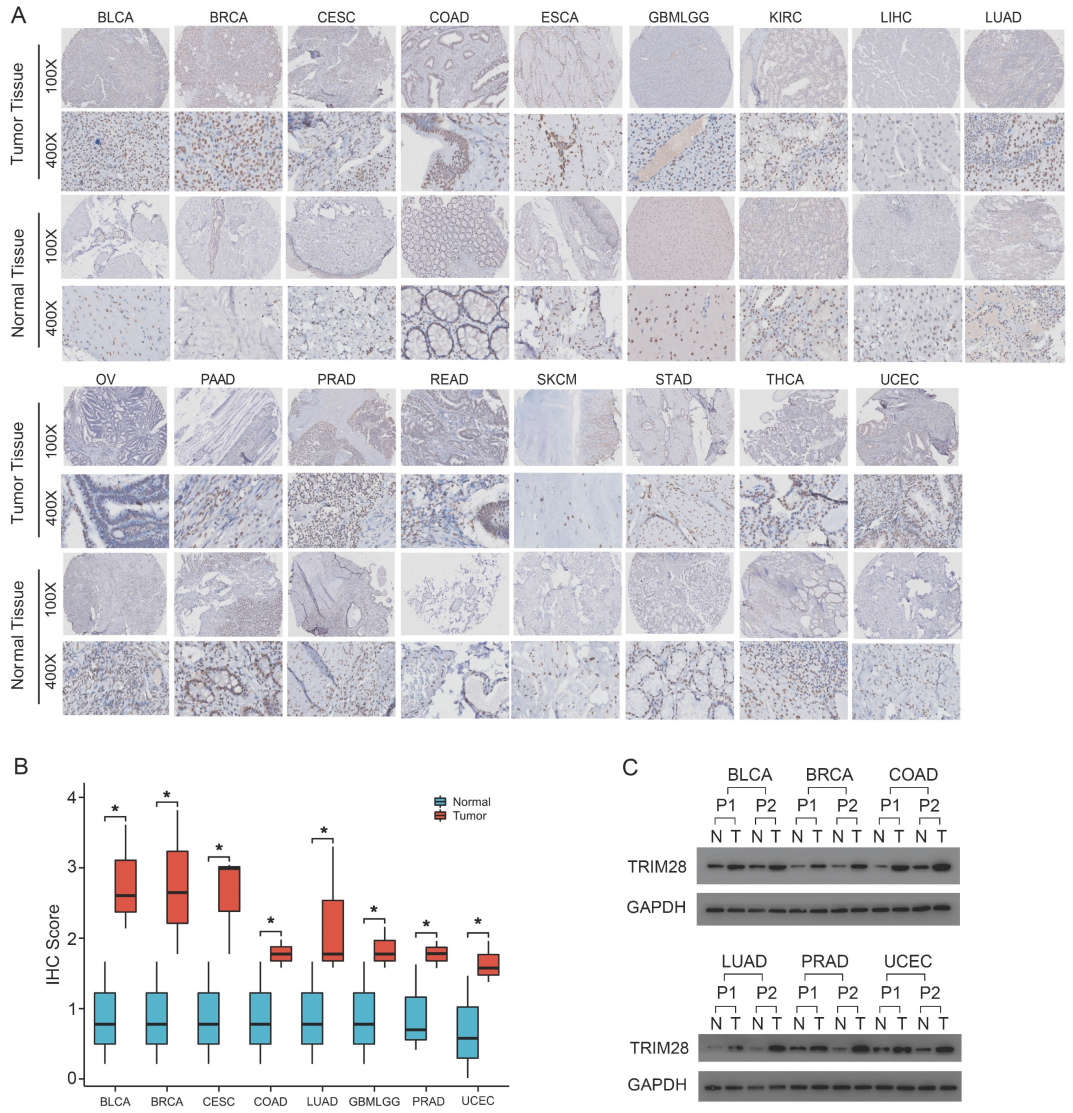
**TRIM28 expression detected by tissue microarray in tumor tissues and normal tissues**. **(A)** BLCA (n=16), BRCA (n=12), CESC (n=22), COAD (n=12), ESCA (n=10), GBMLGG (n=5), KIRC (n=18), LIHC (n=6), LUAD (n=16), OV (n=3), PAAD (n=5), PRAD (n=37), READ (n=32), SKCM (n=2), STAD (n=6), THCA (n=13), and UCEC (n=12). **(B)** Quantified data (IHC score) in BLCA, BRCA, CESC, CLAD, LUAD, GBMLGG, PRAD, and UCEC. **(C)** TRIM28 expression in paraffin-embedding tissues detected by western blot assays in cancer patients with BLCA, BRCA, COAD, LUAD, PRAD, or UCEC. P, patient. *p < 0.05.

To further detect and verify the actual protein expression of TRIM28 in tissues, we tested multiple tumor tissues using tissue microarray. As shown in Figure 2A, we found that as comparing with adjacent normal tissues, TRIM28 protein expression was stronger in most tumor tissues, which was consistent with the data from TCGA and GTEx databases. Meanwhile, a strong staining intensity of TRIM28 protein expression was mainly found in both normal and tumor tissues of KIRC, LIHC, STAD, and ESCA. Also, we found that TRIM28 protein expression level was less in adjacent normal tissues than in tumor tissues of OV, PAAD, and SKCM. We further quantified the IHC data and showed that TRIM28 expression were higher in tumor tissues than that in adjacent normal tissues in BLCA, BRCA, CESC, COAD, LUAD, GBMLGG, PRAD, and UCEC (Figure 2B). Moreover, we conducted the western blotting using paraffin-embedded tumor tissues and verified the higher expression of TRIM28 in tumor tissues than that in normal tissues in BLCA, BRCA, COAD, LUAD, PRAD, and UCEC (Figure 2C). Taken together, these results indicate that TRIM28 is carcinogenic and contributes to cancer progression.

### Prognostic value of TRIM28 across cancers

**Figure 3:**
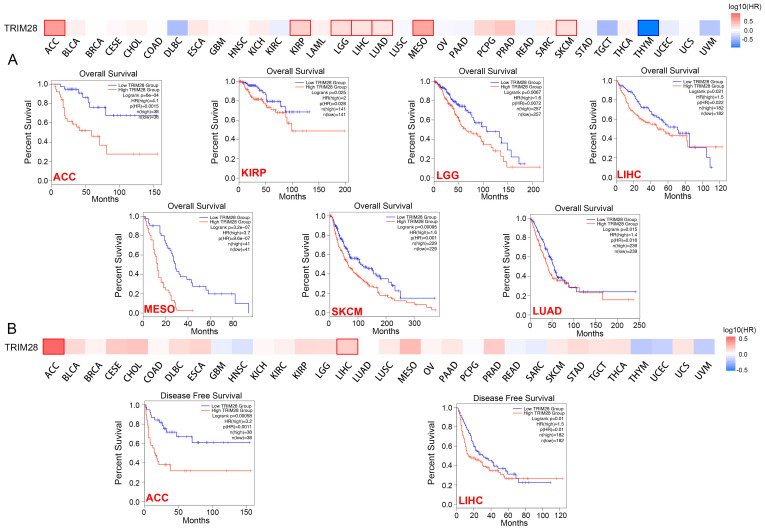
**Correlation between TRIM28 expression and overall survival and disease-free survival in patients with different types of cancer. (A)** Survival curve (OS) from GEPIA2 (upper panel). Kaplan-Meier survival curves (OS) of patients with high and low TRIM28 expression in different types of cancers (ACC, KIRP, LGG, LIHC, MESO, SKCM, LUAD) (lower panel). **(B)** Survival curves (DFS) from GEPIA2 (upper panel). Kaplan-Meier survival curves (DFS) of patients with high and low TRIM28 expression in different types of cancers (ACC, LIHC) (lower panel).

We analyzed the OS, DFI, DSS, and PFI probabilities across cancers to determine whether TRIM28 expression is linked to cancer prognosis. We found that the expression level of TRIM28 was correlated with OS in MESO (*p* = 2.9e-6), ACC (*p* = 1.4e-5), LGG (*p* = 8.5e-5), and LIHC (*p* = 8.6e-5) via Cox proportional hazards analysis (Figure S1). To compare TRIM28's predictive value in human cancers, the difference in prognosis between the high and low TRIM28 expression groups was assessed via the GEPIA2 webtool. The results showed that the OS time of high TRIM28 expression group was significantly shorter than that of the low TRIM28 expression group in ACC (*p* = 6e-04), KIRP (*p* = 0.025), LGG (*p* = 0.0067), LIHC (*p* = 0.021), MESO (*p* = 3.2e-07), SKCM (*p* = 0.00095), and LUAD (*p* = 0.015) (Figure 3A). Simultaneously, higher TRIM28 expression was correlated with a shorter DFS time in ACC (*p* = 0.00058) and LIHC (*p* = 0.01) (Figure 3B). These data suggest that TRIM28 expression is associated with poor prognosis in cancer patients.

### *TRIM28* genetic alterations across cancers

**Figure 4:**
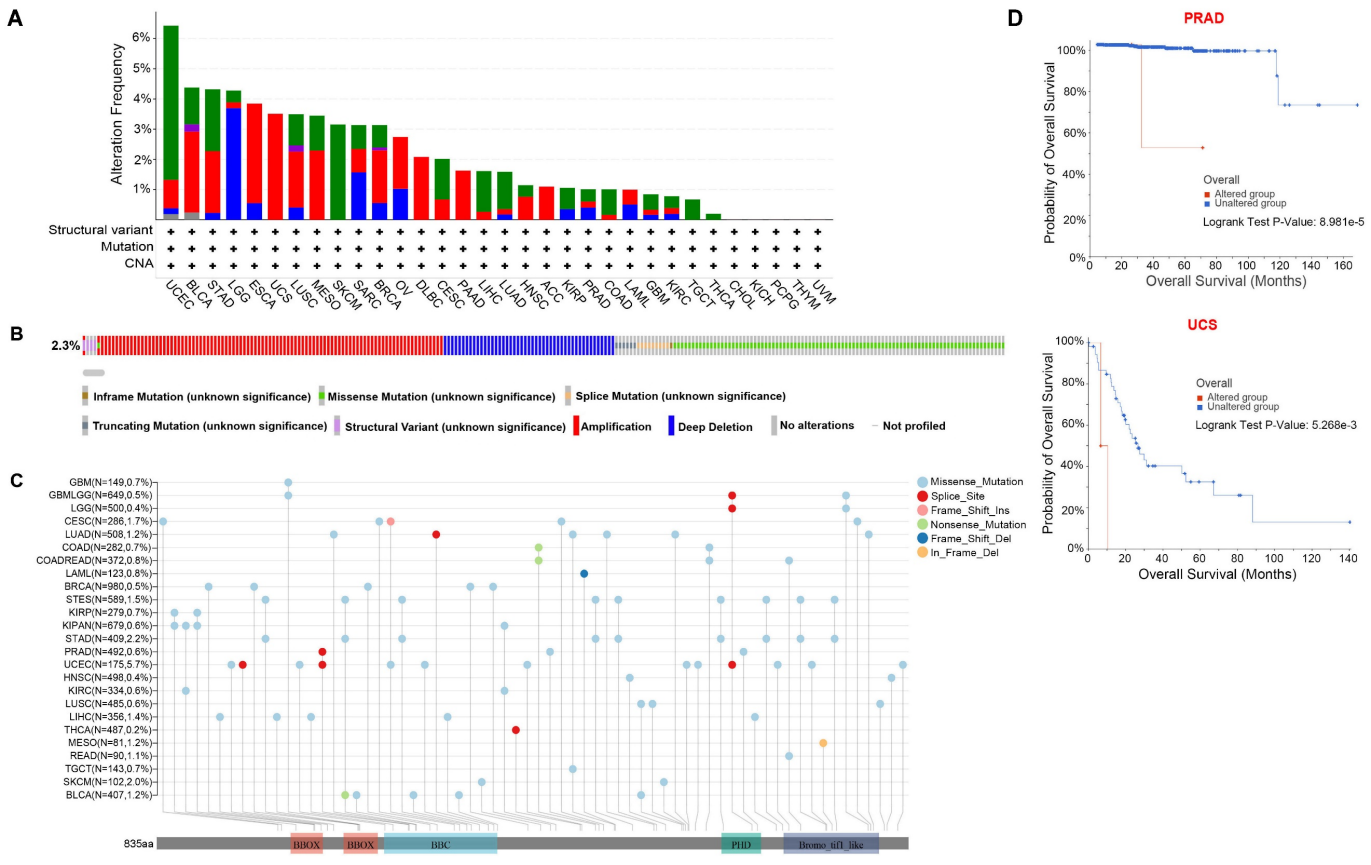
**Analysis of *TRIM28* genetic alterations in different types of cancer. (A)** Frequencies and types of genetic alterations in *TRIM28* gene. **(B)** Oncoprint of *TRIM28* gene alterations in cancer cohorts. **(C)** Mutation sites of the *TRIM28* gene in TCGA samples. **(D)** Kaplan-Meier survival curves (OS) for the altered and unaltered groups in PRAD and UCS.

With cBioPortal (http://www.cbioportal.org), we investigated *TRIM28* genetic alterations in various cancers, and we discovered that UCEC had the most pronounced genetic alterations. Gene mutation and amplification are the most common genetic alterations in *TRIM28* in cancers. In addition, the rate of *TRIM28* genetic alteration was high in UCEC, BLCA, STAD, and LGG, and structural variation was the rarest type of genetic alteration (Figure 4A). Furthermore, the major genetic alterations in the *TRIM28* gene were missense mutation, amplification, and deep deletion (Figure 4B), and the integrated *TRIM28* mutation data in the pan-cancer context are presented in Figure 4C. Moreover, we analyzed the relationships between *TRIM28* genetic alterations and clinical prognosis and found that the OS time of the altered group was considerably shorter than that of the unaltered group in PRAD (*p* < 0.001) and UCS (*p* < 0.001), indicating that *TRIM28* genetic alteration is related to worse prognosis (Figure 4D).

### Correlation between TRIM28 expression and immune cell infiltration

**Figure 5:**
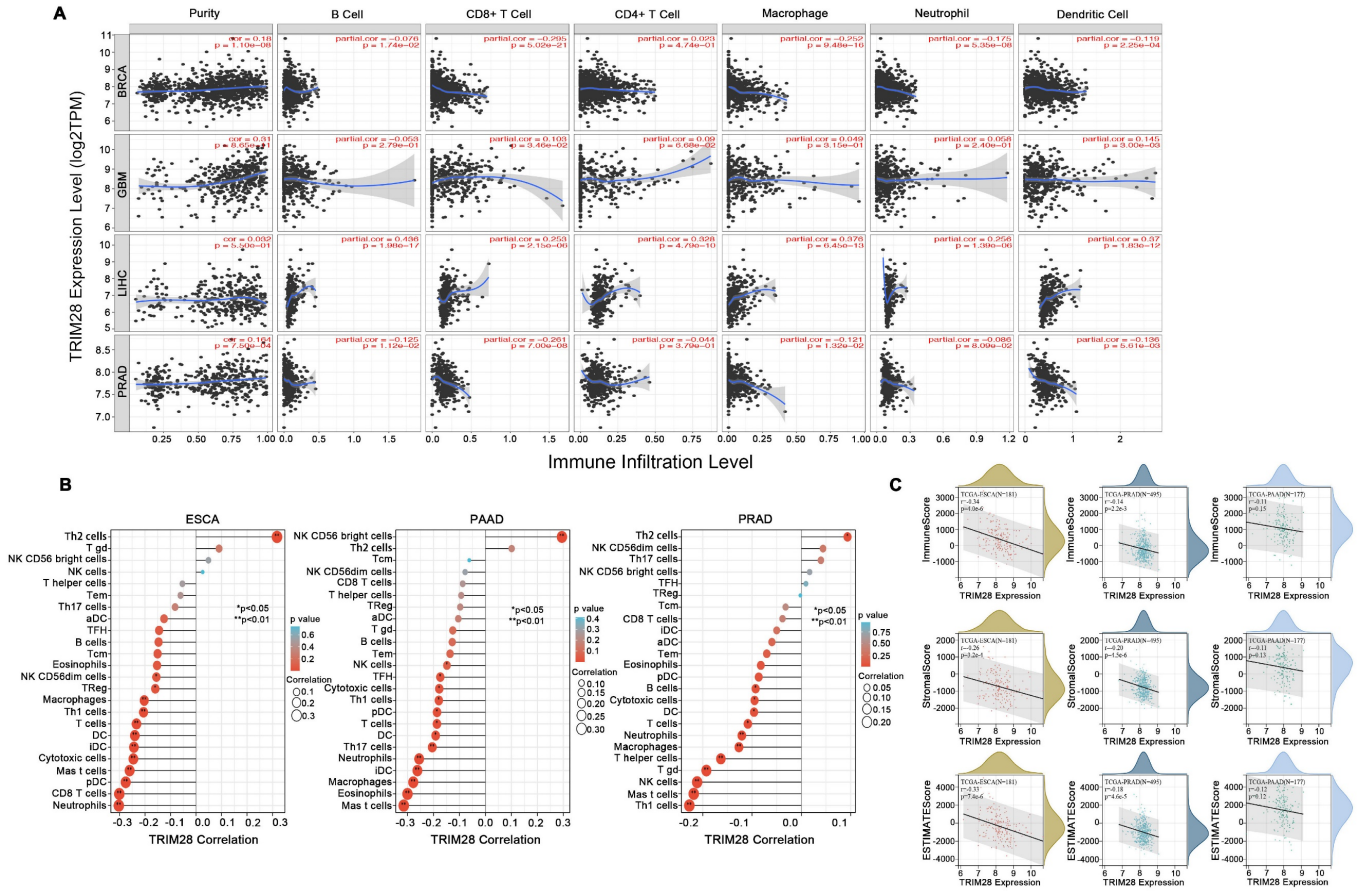
**Correlation between TRIM28 expression and immune infiltration levels in different types of cancer. (A)** The correlations between TRIM28 expression and immune infiltration in BRCA, GBM, LHC, and PRAD. **(B)** The correlations between TRIM28 expression and immune cells in ESCA, PRAD, and PAAD. **(C)** The correlations between TRIM28 expression and immune, stromal, and ESTIMATE scores in ESCA, PRAD, and PAAD.

Tumor tissues contain stromal cells, fibroblasts, and immune cells, and these cells control tumor microenvironment. Thus, we explored the correlations between TRIM28 expression and immune cell infiltration. We found that TRIM28 expression enhanced immune cell infiltration, especially in BRCA, GBM, LIHC, and PRAD, and that TRIM28 expression levels were positively related to tumor purity (*p* < 0.001) and the presence of CD4+ T cells (*p* < 0.001) in BRCA. Additionally, TRIM28 expression was positively related to tumor purity and the presence of CD8+ T cells, CD4+ T cells, macrophages, neutrophils, and dendritic cells in GBM and LIHC (all *p* values < 0.001). However, the expression of TRIM28 was positively associated only with tumor purity (*p* < 0.001) in PRAD (Figure 5A) and was also related to tumor immune infiltration in PRAD, ESCA, and PAAD (Figure 5B). Furthermore, we observed that TRIM28 expression was associated with markedly decreased immune, ESTIMATE, and stromal scores in PRAD, ESCA, and PAAD (Figure 5C). Collectively, these data show that TRIM28 is intimately associated with tumor immune cell infiltration.

### TRIM28 promotes immunotherapy resistance and immune escape

**Figure 6:**
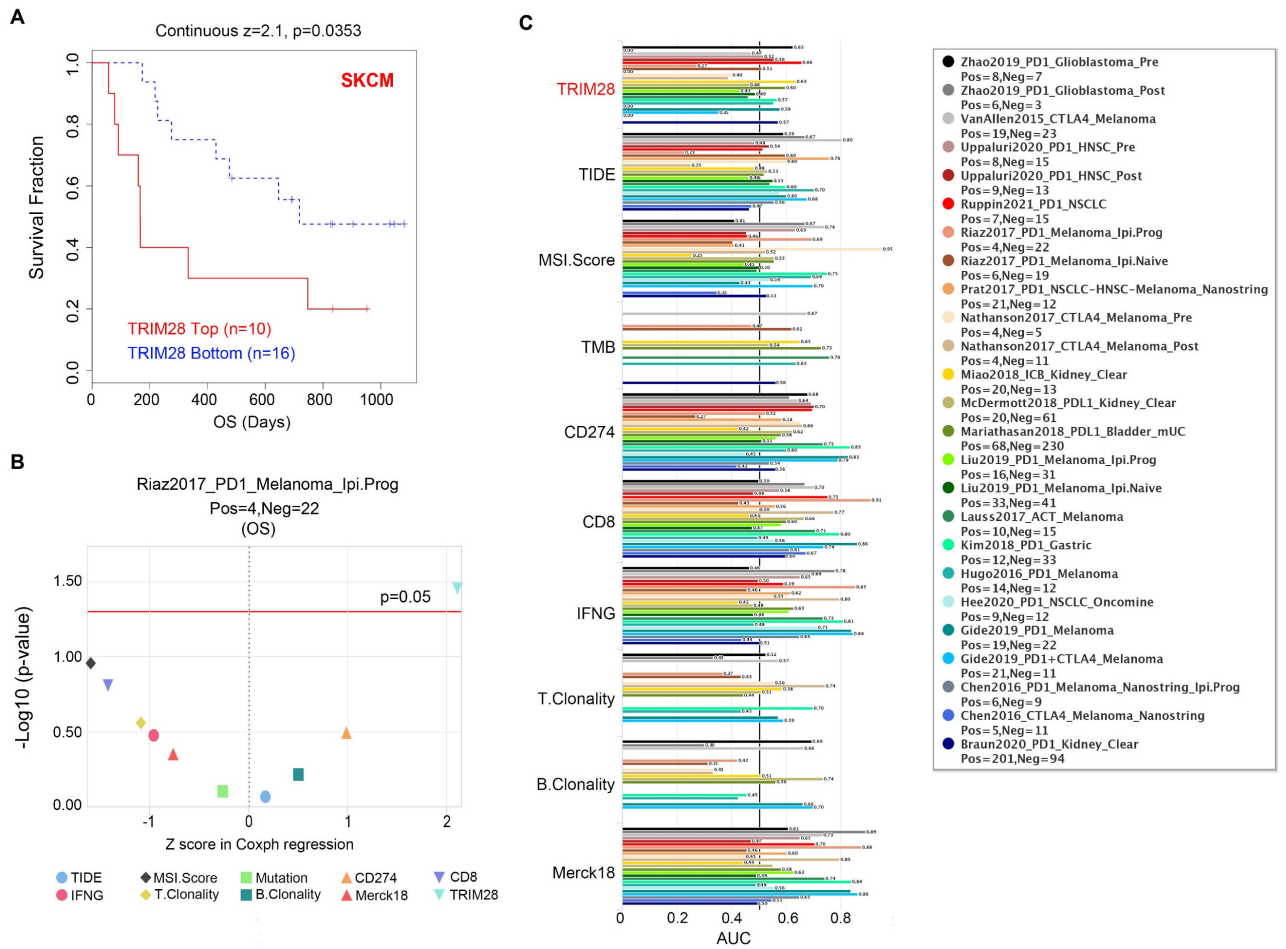
**Correlation between TRIM28 expression and the immunotherapy response. (A)** Kaplan-Meier survival curves (OS) of patients with high and low TRIM28 expression in the SKCM cohort treated with anti-PD1 agents.** (B)** The prognostic value of TRIM28 versus standard biomarkers in the SKCM cohort. **(C)** The correlations between TRIM28 and standard biomarkers in immunotherapy cohorts. The area under the recipient's working characteristic curve (AUC) was used to evaluate the predictive performance of the tested biomarkers for immunotherapy response status.

Blockade of immune checkpoint molecules is essential for the action of immunotherapies. However, quite a few patients show no responses to such immunotherapies. An appropriate and stable indicator that can guide immunotherapy is urgently needed. Due to the close relationship between TRIM28 expression and immune cell inflation, we investigated the potential of TRIM28 as an immunotherapy response biomarker. The results showed that high TRIM28 expression led to worse prognosis and that TRIM28 expression greatly affected the effect of immune checkpoint blockade (anti-PD1) therapy with a decrease in patient OS (*p* = 0.0353) in the Riaz2017_PD1 SKCM cohort (Figure 6A, B). In comparing the predictive ability of TRIM28 to that of standard biomarkers, we found that TRIM28 has advantages in predicting NSCLC patients' immunotherapy outcomes (Ruppin2021_PD1_NSCLC, AUC = 0.66, Figure 6C). These data demonstrate that TRIM28 participates in the antitumor immune response to promote immunotherapy resistance.

**Figure 7:**
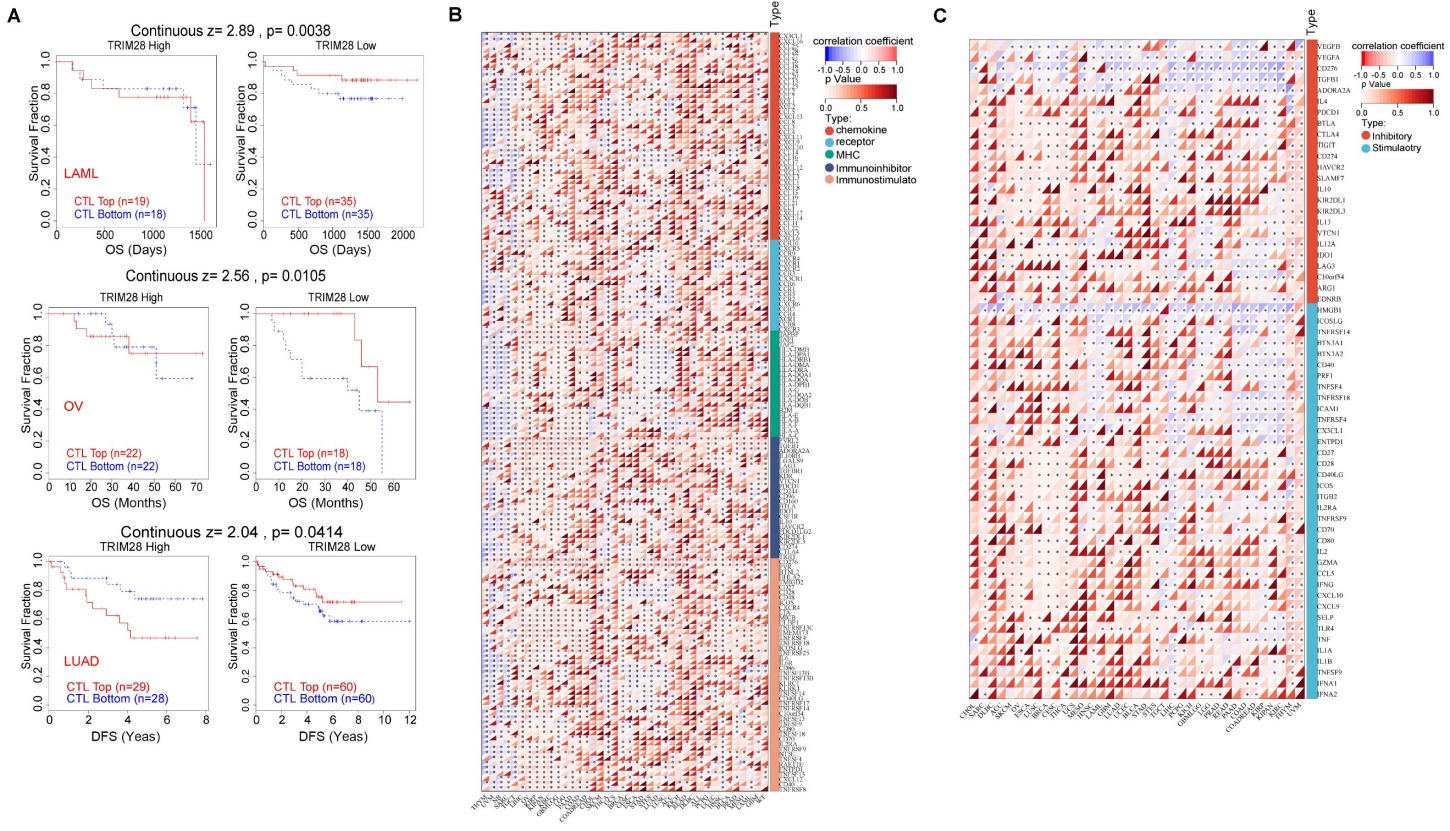
**Mechanism by which TRIM28 promotes immunotherapy resistance. (A)** The relationship between TRIM28 expression and CTL dysfunction in LAML, OV, and LUAD. **(B)** The correlation between the expression of TRIM28 and 150 immunomodulators, including chemokines, receptors, MHC, immunoinhibitors, and immunostimulators. **(C)** The correlations between the expression of TRIM28 and immune checkpoints.

It is widely accepted that CTL dysfunction facilitates tumor resistance to and escape from immunotherapy. We assessed the connection between TRIM28 expression and CTL function via the TIDE database and found that the CTL dysfunction level was positively correlated with the TRIM28 expression level in LAML (*p* = 0.0038), OV (*p* = 0.0105), and LUAD (*p* = 0.0414). In patients with LAML, OV, or LUAD, CTL infiltration was beneficial in those with low TRIM28 expression. Consistent with this finding, when TRIM28 expression was high, the effect of CTL infiltration was reduced or even reversed (Figure 7A). The immunological effects of TRIM28 on cancers are crucial to determine which cancers may benefit from anti-TRIM28 immunotherapy on a pan-cancer basis. We revealed that TRIM28 expression was negatively associated with most of the 150 immunomodulators in BLCA (Figure 7B) and was connected to immune checkpoint (ICP) genes in cancers. To explore TRIM28's potential as an immunotherapy target, we examined the association between its expression and that of ICP genes. For 60 ICP genes, namely, 24 inhibitory and 36 stimulatory genes, a substantial correlation with TRIM28 expression was observed in various cancers, such as UVM, THYM, THCA, SKCM, LIHC, LGG, KIRP, and BRCA (Figure 7C). These data indicate that TRIM28 is involved in immune escape and demonstrate the potential of TRIM28 as an immunotherapy target.

### TRIM28 expression level at the single-cell level

**Figure 8:**
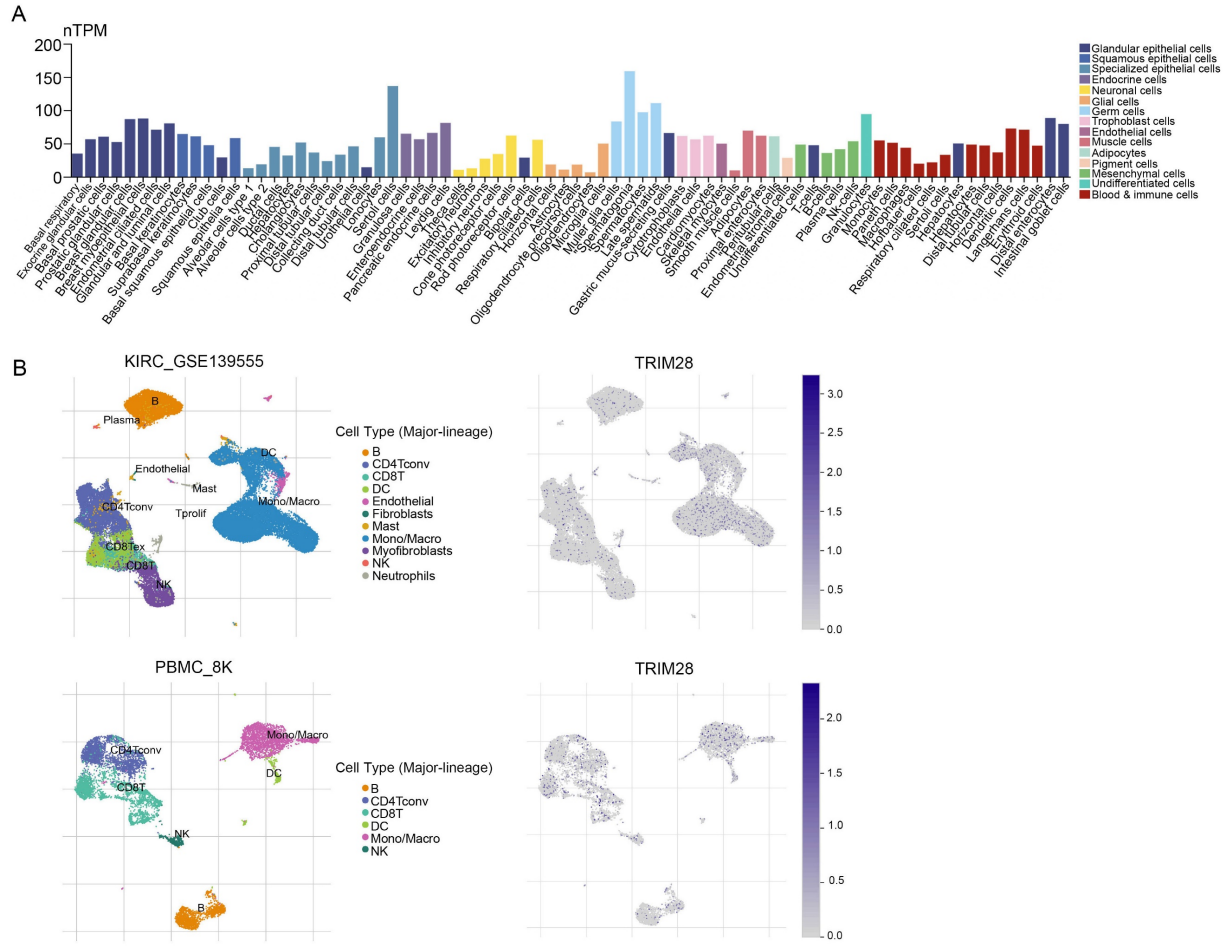
**TRIM28 expression at the single-cell level. (A)** Summary of single-cell RNA data from all cell types. **(B)** UMAP plots showing cell clusters and TRIM28 expression levels in different cell types in KIRC and PBMC.

To test the heterogeneity of TRIM28 expression in different cells, we summarized the RNA expression levels found in single cells (nTPM) of numerous types. By the color code, the cell types were grouped into different functional groups based on their functional characteristics (Figure 8A). To investigate the underlying mechanisms by which TRIM28 influences the TIME, single-cell RNA-seq (scRNA) data were used to evaluate TRIM28 expression in different immune cells. We observed that TRIM28 was principally expressed in monocytes/macrophages, especially in KIRC, and in PBMC (Figure 8B).

### Correlation between *TRIM28* coexpression networks and the immune response

**Figure 9:**
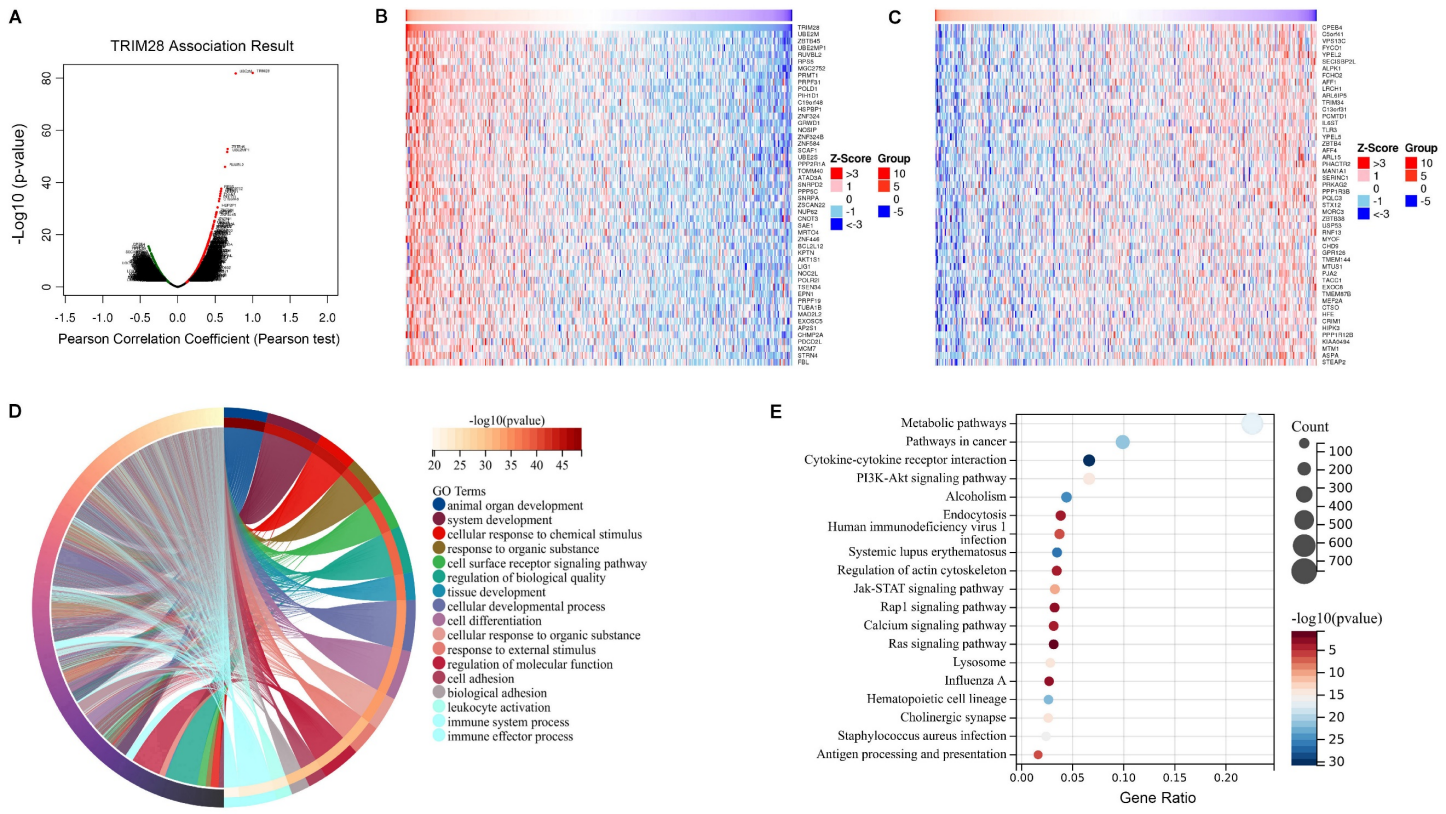
**Analysis of *TRIM28* coexpressed genes in BLCA by the LinkedOmics database. (A)** Genes highly correlated with *TRIM28* were tested by Pearson correlation analysis in the BLCA cohort. **(B)** Heatmap showing the top 50 genes positively coexpressed with *TRIM28* in BLCA. **(C)** Heatmap showing the top 50 genes negatively coexpressed with *TRIM28* in BLCA. **(D)** Chordal graph of TRIM28 GO analysis (biological process) results in the BLCA cohort. **(E)** Bubble plot of TRIM28 KEGG pathway analysis results in the BLCA cohort.

Our results suggested that TRIM28 is significantly associated with cancer prognosis and immunity. To investigate *TRIM28* coexpression networks, we examined the potential mechanism of the *TRIM28* gene in cancer tissues via the LinkedOmics database, and BLCA was chosen as an example to demonstrate the potential effect. The results showed that the expression of 2016 genes (dark red-labeled) was positively associated with that of *TRIM28* and the expression of 2267 genes (dark green-labeled) was negatively associated with that of *TRIM28* in BLCA (Figure 9A). Furthermore, we showed the top 50 genes positively and negatively correlated with *TRIM28* on heatmaps (Figure 9B, C). Among coexpressed genes, the expression of *UBE2M* and *ZBTB45* genes had the closest correlation with *TRIM28* expression (r = 0.774, and 0.666 and *p* = 1.63E-82, and 1.33E-53, respectively). Next, we explored the GO biological process categories and found that the *TRIM28* gene and its coexpressed genes were involved in the immune system process and immune effector process (Figure 9D). Kyoto Encyclopedia of Genes and Genomes (KEGG) pathway analysis indicated that the coexpressed genes were enriched in the cytokine-cytokine receptor interactions and antigen processing and presentation pathways (Figure 9E). Based on these results, TRIM28 may exert important effects on human cancers by modulating immune responses.

### Correlations between TRIM28 expression and microsatellite instability (MSI), neoantigens, and tumor mutational burden (TMB)

**Figure 10:**
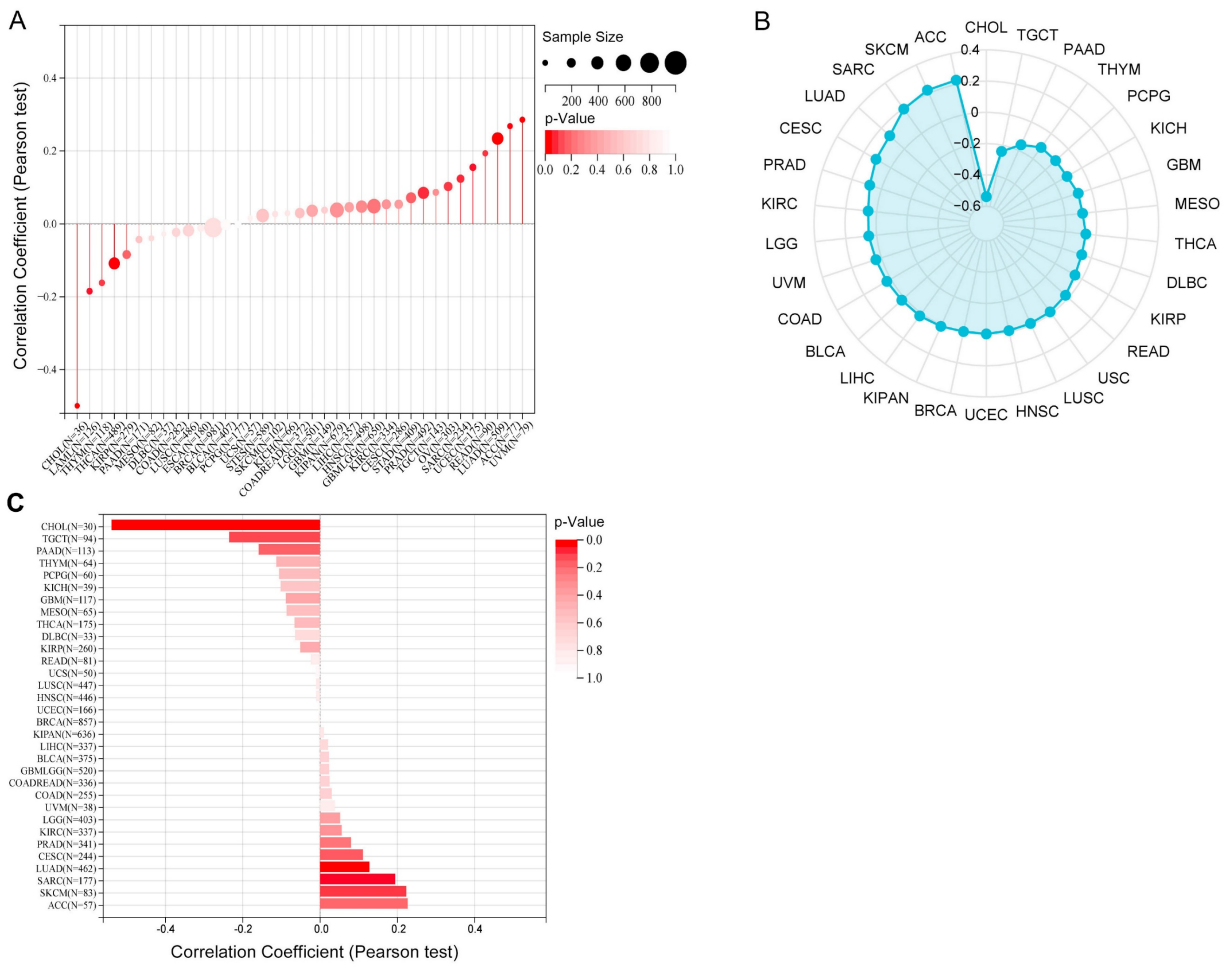
**Relationships between TRIM28 expression and the TMB, MSI and neoantigens. (A)** TMB. **(B)** MSI. **(C)** Neoantigens in human cancers.

MSI, TMB, and neoantigens in the tumor microenvironment are associated with antitumor immunity and may be predictive of the effectiveness of tumor immunotherapy. Our results revealed that TRIM28 expression had strong positive associations with the TMB and MSI in CESC, LUAD, SARC, KIRC, LUSC, and LIHC and negative associations in GBMLGG and THCA (Figure 10A, B). In addition, we found that TRIM28 expression was positively associated with neoantigens in LUAD and SARC and negatively associated with neoantigens in CHOL (Figure 10C). Based on these results, we strongly believe that TRIM28 regulates the tumor microenvironment composition and immune response, thereby affecting the response to antitumor immunotherapy.

## Discussion

Pan-cancer analysis offers comprehensive insights into molecular aberrations in various cancers and helps identify biomarkers for early cancer detection and targeted therapies. According to the TCGA project, 33 types of prevalent tumors have been profiled by a multiomics approach, providing an unprecedented opportunity to discover molecular aberrations across cancer types 20, 21. Due to the development of the TCGA database and bioinformatics techniques 22, 23, the identification of molecular biomarkers and their functions in pan-cancer has become the subject of numerous studies. Our study aimed to determine whether *TRIM28* is a possible oncogenic target gene with important implications for immunotherapy in a variety of cancers.

As a regulatory factor, TRIM28 has been reported to mediate the regulation of multiple immune cell activities and the expression of various cytokines 24-27. To investigate the role of TRIM28 in tumors, we first analyzed its expression in 33 types of cancer, which revealed a widespread overexpression of TRIM28 across cancers. Furthermore, TRIM28 expression was correlated with the classification of cancers such as ACC, KICH, KIRC, KIRP, LIHC, and LUSC. Prognosis analysis revealed shorter OS and DFS times in the high TRIM28 expression group than in the low TRIM28 expression group. Although an increasing number of studies have examined the relationship between gene mutation and the progression of human cancers 28, only a few alterations have been found to markedly influence cancer progression 29. Survival analysis showed that TRIM28 alterations reduced the OS time in PRAD and UCS. However, the mechanism by which TRIM28 alterations affect the prognosis of human cancers has not been examined. In this study, our results provided a basis for a more detailed investigation of the carcinogenic effect of TRIM28.

In addition to regulating tumor biology, TRIM28 was also found to have important implications for immunotherapy 30. We found that the expression of TRIM28 was markedly positively correlated with the presence of T cells, neutrophils, and macrophages. In previous studies, macrophages have been linked to poor prognosis in the early stage of LUAD 31. Furthermore, TRIM28 may influence immune, ESTIMATE, and stromal scores in a variety of cancers. Based on this evidence, it appears that immune infiltration influences cancer progression caused by TRIM28, since we found that TRIM28 was mainly expressed in monocytes/macrophages. Although anti-programmed death 1 (PD-1) and anti-PD-L1 agents have shown significant clinical efficacy and lasting responses with minimal side effects 32, 33, many patients with cancer may not benefit from them, or they may not exhibit ideal efficacy 34. According to GO biological process analysis, the *TRIM28* gene and its co-expressed genes participate in the immune system and immunotherapy resistance as well as leukocyte activation. Furthermore, KEGG analysis revealed that the *TRIM28* coexpressed genes likely participate in antigen presentation and processing. Collectively, these findings indicate that TRIM28 expression appears to exert important effects on human cancers by modulating the immune response in the TIME, but its detailed functions are still unclear. The effect of TRIM28 on the TIME remains to be further studied.

This study has several limitations. First, while our bioinformatics analyses provided insights into TRIM28's impacts on cancer progression and immunotherapy resistance, *in vitro* and *in vivo* biological experiments were lacking. Second, the effects of TRIM28 on different cancers are heterogeneous, and the causes of this heterogeneity should be further identified to help provide accurate and personalized cancer treatment.

## Conclusion

In summary, our findings in this study elucidated the close connections between TRIM28 expression and immune cell infiltration, immune responses, immunotherapy resistance, the TMB, MSI, and neoantigens in a variety of human cancers, indicating that TRIM28 is a potentially valuable immune response indicator and a molecular biomarker for predicting the prognosis of cancer patients.

## Supplementary Material

Supplementary figure.

## Figures and Tables

**Table 1:** Abbreviation of 33 cancer types

Abbreviation	Full name
ACC	Adrenocortical carcinoma
BLCA	Bladder urothelial carcinoma
BRCA	Breast invasive carcinoma
CESC	Cervical squamous cell carcinoma and endocervical adenocarcinoma
CHOL	Cholangiocarcinoma
COAD	Colon adenocarcinoma
DLBC	Lymphoid neoplasm diffuse large B-cell lymphoma
ESCA	Esophageal carcinoma
GBM	Glioblastoma multiforme
HNSC	Head and neck squamous cell carcinoma
KICH	Kidney chromophobe
KIRC	Kidney renal clear cell carcinoma
KIRP	Kidney renal papillary cell carcinoma
LAML	Acute myeloid leukemia
LGG	Brain lower grade glioma
LIHC	Liver hepatocellular carcinoma
LUAD	Lung adenocarcinoma
LUSC	Lung squamous cell carcinoma
MESO	Mesothelioma
OV	Ovarian serous cystadenocarcinoma
PAAD	Pancreatic adenocarcinoma
PCPG	Pheochromocytoma and paraganglioma
PRAD	Prostate adenocarcinoma
READ	Rectum adenocarcinoma
SARC	Sarcoma
SKCM	Skin cutaneous melanoma
STAD	Stomach adenocarcinoma
TGCT	Testicular germ cell tumors
THCA	Thyroid carcinoma
THYM	Thymoma
UCEC	Uterine corpus endometrial carcinoma
UCS	Uterine carcinosarcoma
UVM	Uveal melanoma

## Abbreviations

TRIM28: Tripartite motif-containing protein 28
TCGA: The Cancer Genome Atlas
GTEx: Genotype-Tissue Expression
GEPIA2: Gene Expression Profiling Interactive Analysis 2
TIMER: Tumor Immune Estimation Resource
GSEA: Gene Set Enrichment Analysis
TMB: Tumor Mutational Burden
MSI: Microsatellite Instability
TPM: Transcripts Per Million
OS: Overall Survival
DFS: Disease-Free Survival
HR: Hazard Ratio
ESTIMATE: Estimation of Stromal and Immune cells in Malignant Tumor tissues using Expression data
TIDE: Tumor Immune Dysfunction and Exclusion
TISCH: Tumor Immune Single-Cell Hub
ICP: Immune Checkpoint
scRNA: Single-cell RNA-seq
GO: Gene Ontology
KEGG: Kyoto Encyclopedia of Genes and Genomes
TME: Tumor Microenvironment
