# Supplementary figures and images for: A systematic pan-cancer analysis identifies TRIM28 as an immunological and prognostic predictor and involved in immunotherapy resistance
Source: J Cancer. 2023 Sep 4;14(15):2798–810. doi: 10.7150/jca.86742 (PMC10539564; doi:10.7150/jca.86742)

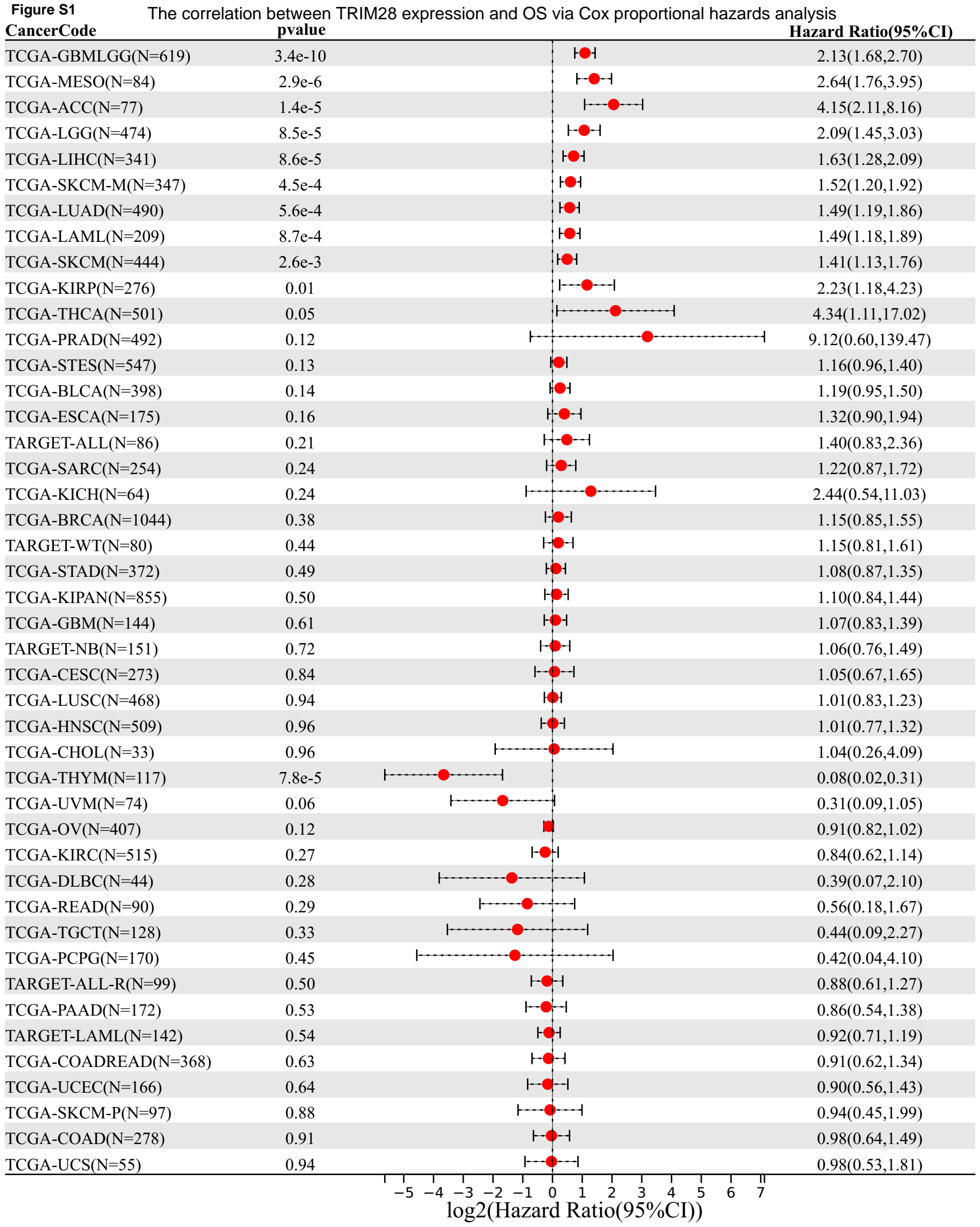

Supplement: Supplementary file 1 — Supplementary figure. [file jcav14p2798s1.pdf]
